# Complications and risk factors of endoscopic third ventriculostomy: A 10-year single-centre study and systematic literature review

**DOI:** 10.1016/j.bas.2025.104291

**Published:** 2025-06-01

**Authors:** Einar Naveen Møen, Christian André Helland, Rupavathana Mahesparan

**Affiliations:** a Department of Clinical Medicine, University of Bergen, Bergen, Norway; b Department of Neurosurgery, Haukeland University Hospital, University of Bergen, Bergen, Norway

**Keywords:** Hydrocephalus, Endoscopic third ventriculostomy, Surgical outcomes, Quality assurance, Systematic review, Case series

## Abstract

**Introduction:**

Endoscopic third ventriculostomy is considered a safe and low-risk treatment of obstructive hydrocephalus. No systematic review has been conducted recently to establish benchmarks for success and complication rates. This knowledge gap makes it difficult to evaluate local institutional performance against international results.

**Research question:**

What are the results of endoscopic third ventriculostomy for obstructive hydrocephalus in the literature, and how does it compare to the results of our medium-sized neurosurgical center?

**Material and methods:**

We performed a retrospective case series of patients treated at the Department of Neurosurgery, Haukeland University Hospital, from January 1, 2013, to December 31, 2023. A systematic review was performed in accordance with the PRISMA guidelines.

**Results:**

Our case series consisted of 127 patients in a mixed-age cohort (mean: 37.3, range: 0–86) treated with endoscopic third ventriculostomy for hydrocephalus the last ten years. Previous shunting and neurosurgery were identified as risk factors for endoscopic third ventriculoscopy failure. In our systematic review, we found 64 reports with a total of 8409 patients eligible for inclusion. A higher success rate (78.7%) and complication rate (21.3%) were found in our patient material compared to the findings in our systematic review (respectively 73.4% and 11.6%). All complications in our material were transient and did not cause any permanent morbidity.

**Discussion and conclusions:**

Endoscopic third ventriculostomy has a favorable safety profile with high success rates in the treatment of obstructive hydrocephalus. Results from our systematic review can be used for internal audits at other neurosurgical centers.

## Highlights

• This is the second literature review on endoscopic third ventriculostomy (ETV) for hydrocephalus since 2011.
• Results from our medium-sized neurosurgical center are comparable to what is reported previously in the literature.
• We found a mean success rate of 73.4% (1%–64%) and complication rate of 11.6% (41%–95%) in our systematic review.
• Previous neurosurgery and previous shunting was identified as risk factors for ETV-failure of endoscopic third ventriculostomy in our series.
• Results from our systematic review provide neurosurgical centers with information for internal audits.

## 1 Introduction

Hydrocephalus is one of the most common pathologies treated in neurosurgical centers. The condition is characterized by excessive accumulation of cerebrospinal fluid, followed by neuronal damage because of increased intracranial pressure (Dewan et al., 2019; Koleva and De Jesus, 2024). Contemporary classifications recognize additional subtypes, but for practical purposes, hydrocephalus can be broadly categorized as communicating, normal-pressure, hypersecretory or obstructive (Koleva and De Jesus, 2024). Treatment options depend on the etiology and include ventriculoperitoneal shunting, endoscopic third ventriculostomy, and choroid plexus coagulation (Koleva and De Jesus, 2024). Ventriculoperitoneal shunting, commonly used for communicating and normal-pressure hydrocephalus, is associated with a complication rate of around 30 % due to its reliance on implants (Ayogu et al., 2024; Dakurah et al., 2016; Hamdan, 2018; Komolafe et al., 2008; Merkler et al., 2017; Morina et al., 2013; Pan, 2018; Wu et al., 2007). Endoscopic third ventriculostomy, which has emerged as the preferred treatment of obstructive hydrocephalus, eliminates the need for implants by leveraging the natural cerebrospinal fluid drainage pathways of the brain (Jones et al., 1990).

Any intervention involving the brain carries inherent neurological risk. While ETV is an established method and recognized among clinicians as a safe, effective, and low-risk procedure for managing obstructive hydrocephalus, some aspects remain uncertain. For instance, postoperative complication rates and severity vary notably between series, with one systematic review from 2011 reporting rates between 2.9% and 16.1% (Bouras and Sgouros, 2011). Success rates also vary considerably across studies, making it difficult to establish clinical performance benchmarks for internal audits, quality assurance and patient counseling. These considerations highlight the value of analyzing institutional outcomes within the context of contemporary literature.

The primary objective of this study was to evaluate the success rate and severity of complications in patients treated with endoscopic third ventriculostomy (ETV) for hydrocephalus at the Department of Neurosurgery, Haukeland University Hospital, from January 1, 2013, to December 31, 2023, and to compare these outcomes with those reported in the existing literature. This will provide updated outcome data for institutional benchmarking and quality assurance. To enable meaningful comparison and contextualization, these results will be compared to international findings through a systematic review of the available literature. Our secondary objective is to identify risk factors that contributed to complications and failure of ETV in our patient series.

## 2 Methods

### 2.1 Study design

The present study has a combined study design, consisting of both a retrospective single-center case series, and a systematic review. The case series consists of all patients who have undergone ETV for hydrocephalus at the Department of Neurosurgery, Haukeland University Hospital between January 1, 2013, and December 31, 2023. Data from this patient series was supplemented with a systematic review conducted in accordance with the Preferred Reporting Items for Systematic Reviews and Meta-Analyses (PRISMA) guidelines (Page et al., 2021). This allowed for comparison with our findings and provided an updated summary of the current knowledge since the last review in 2011 (Bouras and Sgouros, 2011).

### 2.2 Eligibility criteria

All patients who were treated at the Department of Neurosurgery at Haukeland University Hospital with ETV for hydrocephalus between January 1, 2013, and December 31, 2023, were included in our retrospective analysis. Patients with unavailable medical records were excluded.

The aim of our systematic review was to identify and characterize postoperative complication rates and complication types of ETV for hydrocephalus. Thus, reports that fulfilled the following criteria were included:

1. report results of ETV for hydrocephalus,
2. reports at least a 30-day follow-up period, and
3. reports at least three distinct complication types.

Furthermore, reports that fulfilled the following criteria were excluded:

1. reports in languages besides English,
2. reports doing several procedures simultaneously (e.g choroid plexus cauterization together with ETV), and
3. reports with fewer than 10 patients.

### 2.3 Data collection

In our retrospective analysis, we collected data from electronic medical records on descriptive characteristics, medical history, 30-day quality indicators, complications, and outcome data from the first and last follow up. The full extraction sheet with all variables is provided in Appendix 1.

The search strategy for our systematic review was constructed using a trial-and-error approach against an existing sample to optimize the search syntax. The final search syntax is provided in Appendix 2. Using the Ovid interface, we searched Embase and Medline on October 3, 2024. Data on the following items was collected from identified reports: last name of the first author, year, title, center, study type, number of patients, percentage of females, background of population, age, success rate, complication rate, permanent morbidity, procedural mortality, and the counts of all complications that were reported.

### 2.4 Statistical analyses

Statistical analysis was conducted in RStudio using the R programming language. Missing data was excluded from the analyses to maintain the integrity of our analyses within the constraints of our sample size. Considering that sex-specific differences in neurosurgery are largely underexplored, sex was used as a stratification variable in accordance with the Sex and Gender Equity in Research-initiative (Heidari et al., 2016). Fischer's exact test and Welch's *t*-test were used to investigate differences in proportions and averages, respectively, between males and females.

To identify risk factors for complications and failure of ETV (defined as subsequent shunting), we used multivariable Firth's bias-reduced logistic regression. Assumed risk factors were used as independent variables in both models. Prevalence of complications and failure of ETV was used as dependent variables in their respective models. Survival time of ETV in patients with no prior shunting was illustrated using a Kaplan-Meyer plot. Due to quasi-complete separation, previous shunting was analyzed descriptively using Fischer's exact test rather than included in the multivariable logistic regression.

### 2.5 Qualitative analyses

As outlined in the PRISMA-guidelines, a systematic review should include a risk of bias assessment and certainty of evidence assessment (Page et al., 2021). Recent evidence suggests large language models can perform risk of bias assessments with reasonable accuracy (Lai et al., 2024). Based on this evidence, we employed Claude 3.5 from Anthropic to conduct risk of bias assessments that was subsequently validated by the authors. The following tools were used, depending on the study type:

1. Risk of Bias in Nonrandomized Studies of Interventions (ROBINS-I) (Sterne et al., 2016),
2. Risk of Bias in Randomized Studies (RoB) (Sterne et al., 2019), and
3. Joanna Briggs Institute Critical Appraisal Checklist for Case Series (JBI) (Munn et al., 2020).

We were unable to conduct a certainty evidence-assessment as outlined in the PRISMA-guidelines for two reasons. Firstly, most of the identified studies were descriptive, reporting complication and success rates. Such studies are not suitable for Grading of Recommendations, Assessment, Development and Evaluation-analysis (GRADE) (Schünemann et al., 2008) where the objective is to compare interventions for an outcome. Secondly, while a few studies did compare interventions, they did not provide useable effect estimates with confidence intervals. Consequently, a certainty of evidence-assessment could not be carried out.

## 3 Results

### 3.1 Demographics and indications

Between January 1, 2013, and December 31, 2023, 128 patients were treated for hydrocephalus with ETV at the Department of Neurosurgery, Haukeland University Hospital. Concurrent choroid plexus coagulation was not given in addition to ETV. Thirty-four patients (26.8 %) were pediatric cases (<18 years). Because there was a low number of pediatric cases in our material, we decided to analyze adult and pediatric cases together. Records were unavailable for one patient, hence the final review consisted of 127 patients with 72 males and 55 females. The mean age was 37.3 years overall, with males having a mean of 40.5 years and females 33.1 years. Indication varied notably in both pediatric and adult cases. The indication for surgery in pediatric patients was mostly because of different tumors. In adult cases, the indication was often aqueductal stenosis – but there were large variations. Females had a higher prevalence of epilepsy preoperatively, while males reported higher levels of alcohol consumption. No other significant differences between the sexes were observed (Table 1)

**Table 1:** Demographics and clinical characteristics.

Variable	Total (n = 127)	Male (n = 72)	Female (n = 55)	P-value
Background				
average age	37.28	40.50	33.07	0.10
average BMI	25.03	25.42	24.46	0.37
Comorbidities				
diabetes mellitus (n [%])	5 (3.94%)	3 (4.17%)	2 (3.64%)	1.00
pituitary disorders (n [%])	1 (0.79%)	1 (1.39%)	0 (0.00%)	1.00
vision disorders (n [%])	15 (11.81%)	10 (13.89%)	5 (9.09%)	0.58
epilepsy (n [%])	10 (7.87%)	2 (2.78%)	8 (14.55%)	0.02
Immunodeficiency (n [%])	0 (0.00%)	0 (0.00%)	0 (0.00%)	1.00
cardiovascular disorders (n [%])	37 (29.13%)	23 (31.94%)	14 (25.45%)	0.44
Drugs				
anticoagulants (n [%])	13 (10.24%)	10 (13.89%)	3 (5.45%)	0.15
antihypertensives (n [%])	20 (15.75%)	15 (20.83%)	50 (9.09%)	0.09
cholesterol lowering (n [%])	13 (10.24%)	9 (12.50%)	4 (7.27%)	0.39
Stimulants				
smoking (n [%])	22 (18.49%)	11 (20.29%)	11 (16.00%)	0.64
alcohol (n [%])	19 (15.97%)	10 (23.19%)	9 (6.00%)	0.01
Hydrocephalus				
obstructive alone (n [%])	98 (77.17%)	56 (77.78%)	42 (76.36%)	1.00
communicating alone (n [%])	24 (18.90%)	13 (18.06%)	11 (20.00%)	0.82
other types (n [%])	5 (3.94%)	3 (4.17%)	2 (3.64%)	1.00
previously shunted (n [%])	18 (14.17%)	12 (16.67%)	6 (10.91%)	0.45

Table 1. This table reports on the demographics and clinical characteristics across five categories of all patients included in our retrospective analysis in total, and specifically for patients with and without prior shunting. Cases with unavailable data for stimulants and BMI was omitted from the calculations of proportions and averages, respectively. To determine significance in difference in averages, we used the Welch's *t*-test. To determine significance in difference in proportions, we used Fischer's exact test.

### 3.2 Quality indicators and outcomes

Similarly to previous studies on ETV and hydrocephalus, success was defined as subsequent shunt independence at the time of data collection. We found that 27 patients (21.3%) required subsequent shunting, which translates to an overall success rate of 78.7%. Complications occurred in 27 patients (21.3%) – none of which resulted in permanent morbidity. Fourteen patients (11.0 %) were readmitted, and nine patients (7.0%) were reoperated in the 30-day postoperative period. Five patients (3.9%) died from comorbidity in the 30-day post-operative period. No procedure-related deaths were observed (Table 2). There were no statistically significant differences between adult and pediatric cases regarding failure patterns (18% versus 29%; p-value: 0.2) or complications (22% versus 21%; p-value: >0.9).

**Table 2:** Quality indicators.

Indicator	Total (n = 127)	Male (n = 72)	Female (n = 55)	P-value
average length of stay	7.80	8.93	6.33	0.09
subsequent shunting (n [%])	27 (21.26%)	17 (23.61%)	10 (18.18%)	0.52
any complication (n [%])	27 (21.26%)	16 (22.22%)	11 (20.00%)	0.83
30-day reoperation (n [%])	9 (7.09%)	6 (8.33%)	3 (5.45%)	0.73
30-day readmission (n [%])	14 (11.02%)	8 (11.11%)	6 (10.91%)	1.00
30-day mortality overall (n [%])	5 (3.94%)	4 (5.56%)	1 (1.82%)	0.39
30-day procedure-related mortality (n [%])	0 (0.00%)	0 (0.00%)	0 (0.00%)	1.00
30-day nosocomial infection (n [%])	5 (3.94%)	5 (6.94%)	0 (0.00%)	0.07
20-day wound infection (n [%])	5 (3.94%)	2 (2.78%)	3 (5.45%)	0.65
30-day use of external ventricular drain (n [%])	5 (3.94%)	3 (4.17%)	2 (3.64%)	1.00

Table 2. This table reports on the quality indicators in patients included in our retrospective analysis in total and stratified to male and female. To determine significance in difference in averages, we used the Welch's *t*-test. To determine significance in difference in proportions, we used Fischer's exact test.

Most patients (83.3%) experienced a relief in symptoms at the first follow-up, which for most patients was three months after surgery. This was subsequently reflected in decreased size of the ventricular system and patent flow across the stoma on imaging as well (86.1%). Similar results were found at the last follow-up, where 81.1% of patients with available data reported a continued subjective improvement, and imaging showed improvement in 77.8% of available cases (Appendix 4).

### 3.3 Complications and risk factors

The most common complication types were electrolyte disorders (4.7%), cerebrospinal fluid leakage (3.9%), and infections, including nosocomial infection (3.9%), wound infection (3.9%), and other infections (2.4%). As previously mentioned, all complications were transient and left no permanent morbidity. Furthermore, there were no differences in complication types and rates between the two genders, but nosocomial infections were slightly higher in males (p-value: 0.07) (Table 3). Thirteen variables were investigated as potential predictors of complications or failure of ETV (defined as requiring shunting after ETV). No significant predictors of complications were found. Previous neurosurgery besides shunting was identified as a significant predictor of ETV failure (OR: 7.33; 95% CI: 1.94–32.0; p-value: 0.003). Most patients with previous neurosurgery were treated with external ventricular drainage (EVD) (58.3%). Previous shunting was present in 18 of 127 (14.2%) patients requiring VP shunt after ETV and was identified as a significant predictor of ETV failure using Fisher's exact test (OR: 17.0; 95% CI: 4.82–70.71; p-value: <0.001). No other significant variables were found (Table 4).

**Table 3:** Complications.

Complication	Total (n = 127)	Male (n = 72)	Female (n = 55)	P-value
30-day procedure-related mortality (n [%])	0 (0.00%)	0 (0.00%)	0 (0.00%)	1.00
30-day nosocomial infection (n [%])	5 (3.94%)	5 (6.94%)	0 (0.00%)	0.07
20-day wound infection (n [%])	5 (3.94%)	2 (2.78%)	3 (5.45%)	0.65
30-day usage of external ventricular drain (n [%])	5 (3.94%)	3 (4.17%)	2 (3.64%)	1.00
30-day other infection (n [%])	3 (2.36%)	1 (1.39%)	2 (3.64%)	0.58
30-day bleeding (n [%])	2 (1.57%)	2 (2.78%)	0 (0.00%)	0.51
30-day pituitary disorder (n [%])	1 (0.79%)	1 (1.39%)	0 (0.00%)	1.00
30-day electrolyte disorder (n [%])	6 (4.72%)	3 (4.17%)	3 (5.45%)	1.00
30-day vision disorder (n [%])	1 (0.79%)	1 (1.39%)	0 (0.00%)	1.00
30-day dizziness (n [%])	0 (0.00%)	0 (0.00%)	0 (0.00%)	1.00
30-day headache (n [%])	3 (2.36%)	2 (2.78%)	1 (1.82%)	1.00
30-day deep vein thrombosis (n [%])	0 (0.00%)	0 (0.00%)	0 (0.00%)	1.00
30-day seizures (n [%])	0 (0.00%)	0 (0.00%)	0 (0.00%)	1.00
30-day neurological deficit (n [%])	1 (0.79%)	0 (0.00%)	1 (1.82%)	0.43
30-day meningitis (n [%])	1 (0.79%)	1 (1.39%)	0 (0.00%)	1.00
30-day CSF leak (n [%])	5 (3.94%)	4 (5.56%)	1 (1.82%)	0.39
30-day any complication (n [%])	27 (21.26%)	16 (22.22%)	11 (20.00%)	0.83

Table 3. This table reports on complication rates and complication types in patients included in our retrospective review in total, and specifically for patients with and without prior shunting. To determine significance in difference in proportions, we used Fischer's exact test.

**Table 4:** Risk factors for complications and failure.

Characteristic	Complications			Failure		
	OR^a^	95 % CI^b^	p-value	OR^a^	95 % CI^b^	p-value
Birth-Registered Sex	1.15	0.38, 3.51	0.8	0.75	0.19, 2.59	0.7
Age at Surgery	1.02	0.98, 1.05	0.3	1.02	0.99, 1.06	0.3
Body Mass Index	1.05	0.93, 1.17	0.4	0.99	0.87, 1.12	0.9
Diabetes Mellitus	3.71	0.20, 84.9	0.4	3.53	0.29, 46.3	0.3
Vision Disorder	1.63	0.37, 6.03	0.5	1.25	0.26, 5.09	0.8
Epilepsy	1.34	0.18, 7.63	0.8	6.29	0.80, 45.8	0.078
Cardiovascular Disease	0.21	0.02, 1.08	0.063	1.25	0.21, 6.37	0.8
Anticoagulants	7.80	0.56, 196	0.13	0.98	0.09, 9.78	>0.9
Previous Neurosurgery	1.00	0.21, 3.70	>0.9	7.33	1.94, 32.0	0.003
Antihypertensive Medication	0.71	0.03, 10.5	0.8	0.51	0.05, 5.06	0.6
Cholesterol-Lowering Medication	0.14	0.00, 2.03	0.2	5.41	0.53, 56.9	0.15
Smoking	1.82	0.45, 7.19	0.4	0.40	0.06, 2.02	0.3
Alcohol	1.38	0.32, 5.31	0.6	4.23	0.99, 20.0	0.052

Table 4. This table reports on risk factors and predictive factors for respectively complications and failure of endoscopic third ventriculostomy for hydrocephalus using multivariate logistic regression.

a OR = Odds Ratio.

b CI = Confidence Interval.

### 3.4 Results from systematic review

As shown in Fig. 2, 152 reports were sought for full text to assess eligibility. Sixty-four reports were included in the review (20-83), as shown in Table 5. Forty-four reports were retrospective case series (RCS), one report was a retrospective cohort study (RCHS), eleven reports were prospective case series (PCS), three reports were randomized controlled trials (RCT), and five reports were unspecified case series (UCS). Citations for all reports assessed for eligibility are provided in Appendix. The total body of evidence encompassed 8409 patients treated for hydrocephalus with ETV. The studies varied in patient demographics, with 33 studies investigating a mixed population, 15 studies investigating a pediatric population, 11 investigating an adult population, and 3 studies not reporting population age. Of the 6698 patients with reported sex, 2936 (43.8 %) were female (p < 0.001).

**Fig. 1:**
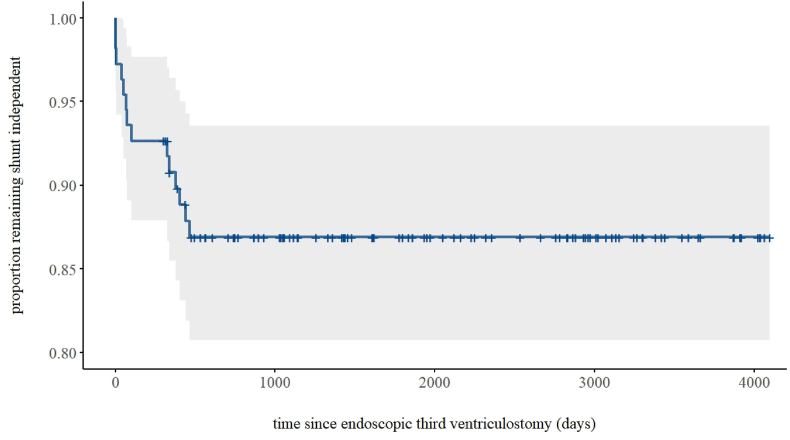
Survival in Endoscopic Third Ventriculostomy For Patients Without Prior Shunting Fig. 1. This figure shows the survival of endoscopic third ventriculostomy in patients with no prior shunting (n = 109).

**Fig. 2:**
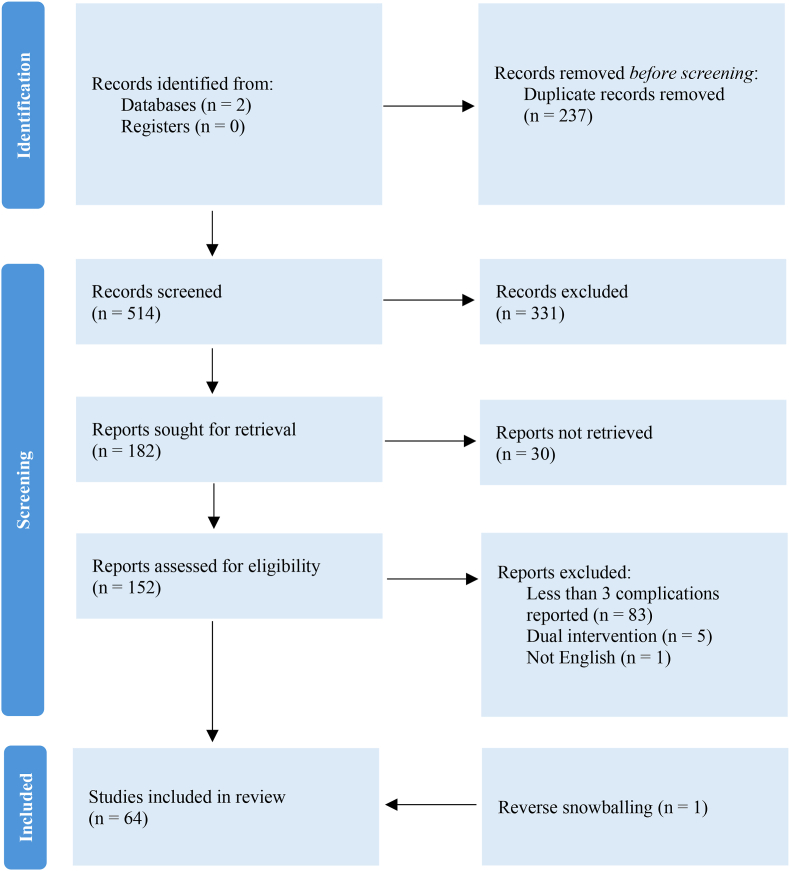
Flow Diagram for Systematic Reviews Fig. 2. This figure shows the process of identifying reports eligible for inclusion in our systematic review.

**Table 5:** Studies included in systematic review.

Study ID	Type	Patients (n)	Females (%)	Age	Success Rate (%)	Complication Rate (%)
Brockmeyer et al., 1998	RCS	97	39 %	mixed	49 %	8 %
Cinalli et al., 1998	RCS	30	30 %	mixed	77 %	13 %
Gangemi et al., 1999	RCS	125	42 %	mixed	86 %	12 %
Hopf et al., 1999	RCS	100	43 %	mixed	76 %	6 %
Fukuhara et al., 2000	RCS	89	49 %	mixed	62 %	8 %
Sainte-Rose et al., 2001	RCS	206	na	mixed	na	29 %
Schroeder et al., 2002	PCS	188	47 %	mixed	66 %	16 %
Siomin et al., 2002	RCS	101	39 %	mixed	na	15 %
Grunert et al., 2003	RCS	159	43 %	mixed	71 %	4 %
Gorayeb et al., 2004	RCS	36	53 %	pediatric	64 %	11 %
Ruggiero et al., 2004	UCS	63	na	pediatric	na	43 %
Kadrian et al., 2005	RCS	203	49 %	na	na	13 %
O'Brien et al., 2005	RCS	233	na	mixed	73 %	5 %
Ray et al., 2005	RCS	43	53 %	mixed	69.80 %	21 %
Santamarta et al., 2005	RCS	66	50 %	adult	71.60 %	9 %
Warf, 2005	PCS	300	44 %	pediatric	59 %	1 %
Navarro et al., 2006	RCS	122	na	pediatric	na	10 %
Ray et al., 2006	RCS	67	52 %	mixed	83 %	19 %
Gangemi et al., 2007	RCS	140	45 %	mixed	na	10 %
Meling et al., 2007	RCS	188	48 %	mixed	na	5 %
Dusick et al., 2008	RCS	108	52 %	adult	na	15 %
Ersahin and Arslan, 2008	RCS	155	45 %	mixed	74 %	15 %
Hader et al., 2008	RCS	131	na	mixed	na	16 %
Kadrian et al., 2008	RCS	203	49 %	mixed	89 %	8 %
Van Beijnum et al., 2008	RCS	202	53 %	mixed	68 %	11 %
Bhatia et al., 2009	RCS	37	na	pediatric	86 %	8 %
Idowu et al., 2009	RCS	29	45 %	pediatric	90 %	10 %
Jenkinson et al., 2009	RCS	190	na	mixed	78 %	6 %
Rahme et al., 2009	RCS	46	37 %	mixed	76.3 %	13 %
Melikian et al., 2010	RCS	60	na	mixed	72 %	20 %
Sacko, et al. 2010	RCS	368	50 %	mixed	69 %	10 %
Naftel et al., 2011	RCS	151	43 %	pediatric	68 %	9 %
Ali et al., 2013	UCS	155	54 %	mixed	71 %	12 %
Chan et al., 2013	PCS	652	41 %	mixed	na	18 %
Vogel et al., 2013	RCS	100	52 %	mixed	75 %	8 %
Bisht et al., 2014	RCS	102	23 %	mixed	82 %	13 %
Goyal et al., 2014	RCT	24	29 %	pediatric	na	na
Grand et al., 2015	RCS	250	na	adult	73 %	4 %
Kawsar et al., 2015	UCS	412	46 %	mixed	78 %	na
Isaacs et al., 2016	RCS	163	44 %	adult	87 %	6 %
Khan et al., 2016	PCS	120	34 %	na	89 %	8 %
Kulkarni et al., 2016	PCS	204	na	pediatric	65 %	na
Sankey et al., 2016	RCS	103	52 %	adult	74 %	9 %
Waqar et al., 2016	RCS	190	na	adult	73 %	6 %
Aref et al., 2017	RCS	131	37 %	adults	95 %	8 %
de Boorder et al., 2018	RCS	106	42 %	mixed	80 %	5 %
Jung et al., 2018	RCS	50	34 %	mixed	80 %	8 %
Uche et al., 2018	PCS	25	na	pediatric	na	20 %
Sarmast et al., 2019	PCS	53	45 %	pediatric	94 %	na
Furtado et al., 2020	RCS	43	54 %	mixed	53 %	9 %
Kontojannis et al., 2020	UCS	20	30 %	adult	89 %	25 %
Arslan et al., 2021	RCS	317	41 %	mixed	72 %	4 %
Chowdhary et al., 2021	PCS	14	29 %	pediatric	79 %	64 %
Faquini et al., 2021	RCS	209	33 %	mixed	83 %	na
Prajapati et al., 2022	RCHS	43	42 %	pediatric	70 %	16 %
Rocque et al., 2022	PCS	203	43 %	pediatric	41 %	22 %
Tabakow et al., 2022	PCS	68	47 %	adult	na	37 %
Ujjan et al., 2022	UCS	97	32 %	mixed	na	23 %
Ul Haq et al., 2022	RCT	35	60 %	na	86.7 %	17 %
Vemula et al., 2022	RCS	50	30 %	mixed	88 %	16 %
Zwimpfer et al., 2022	PCS	42	45 %	adult	na	17 %
Goel et al., 2023	RCS	136	38 %	adult	77 %	4 %
Shinde et al., 2023	RCT	14	29 %	pediatric	71 %	na
Lu et al., 2024	RCS	42	33 %	adult	83 %	31 %

Table 5. This table describes all reports included in our systematic review. We identified 64 studies eligible for inclusion. Most studies were retrospective case-series (RCS), but there were a notable number of studies that were prospective case series (PCS), unspecified case series (meaning that they did not specify whether cases were included prospectively or analyzed retrospectively [UCS]), and randomized controlled trials (RCT). We defined success rate as subsequent shunt independence at the time of follow-up. We defined adults as ≥ 18 years of age, and paediatrics as < 18 years of age.

The risk of bias was evaluated with Claude 3.5 from Anthropic various guidelines depending on the study type. The JBI checklist was used for 60 studies, ROBINS-I for 1 study, and RoB 2.0 for 3 studies. Our assessment identified a low risk of bias in 56 studies, and a moderate risk of bias in 7 studies. The AI model used for assessments demonstrated a high accuracy, with corrections made on 9 judgements.

Data on clinical outcomes were collected for success rates, complication rates, permanent morbidity, mortality, complication type, and complication frequency. This is presented in Table 6. Reports with missing data were excluded from the analyses, meaning that the proportions presented in Table 6 are for the number of patients where the variables were reported. The extent of missing data varied between outcomes and is presented in Appendix 3. A detailed report of complications, risk of bias-assessment, and descriptive statistics on the studies identified in our systematic review is available in Supplementary File 1.

**Table 6:** Results of studies included in systematic review.

Variable	Result
Outcomes	
success rate (% [range])	73.44 % (41 %–95 %)
complication rate (% [range])	11.58 % (1 %–64 %)
permanent morbidity (% [range])	0.74 % (0 %–15 %)
mortality (% [range])	0.89 % (0 %–7 %)
Complication Types	
cardiovascular complications (n [%])	353 (4.20 %)
cerebrospinal fluid complications (n [%])	290 (3.45 %)
infectious complications (n [%])	248 (2.95 %)
neurological complications (n [%])	139 (1.65 %)
technical complications (n [%])	61 (0.73 %)
endocrinologic complications (n [%])	40 (0.48 %)
wound complications (n [%])	37 (0.44 %)
other complications (n [%])	90 (1.07 %)

Table 6. This table presents outcomes and complication types of the studies included in our systematic review. The most common complication types where cardiovascular complications, cerebrospinal fluid complications, and infectious complications. A detailed description of what the complication categories entailed, can be found in Supplementary File 1.

### 3.5 Missing data

Data was collected across 45 domains in our retrospective case-series. There was no missing data in 35 domains. In the remaining ten domains, eight domains had a proportion of missing data greater than 10 %. Missing data was especially prevalent for follow-up variables, including results from imaging and relief in symptoms. In our systematic review, data was missing in five domains. The extent of missing data is reported in detail in Appendix 3.

## 4 Discussion

### 4.1 Clinical outcomes

Our findings demonstrate that endoscopic third ventriculostomy (ETV) can be performed with a high rate of success and a favorable safety profile, even in a medium-sized neurosurgical center. The observed success rate of 78.7 % in our series exceeds the pooled rate of 73.4 % (range: 41 %–95 %) identified in our systematic review, suggesting that outcomes comparable to or better than those in larger centers are achievable with appropriate patient selection and surgical expertise. Notably, we observed no cases of procedure-related mortality or permanent morbidity, while the corresponding rates in the literature were 0.9 % and 0.7 %, respectively. These findings highlight the potential for ETV to be a safe and effective treatment option for hydrocephalus in diverse institutional settings.

However, we found a higher complication rate in our series (21.3%) compared to those found in our systematic review (11.6%; range: 1%–64%). These findings appear contradictory to our lower rates of permanent morbidity and procedural mortality. The discrepancy may reflect differences in practices for defining and documenting complications as well as conducting follow-up consultations. This illustrates the complexity of making comparisons across different institutional settings and highlights the need to examine multiple outcomes when evaluating institutional performance. Additionally, it might also reflect opportunities for refined patient selection criteria at our center, such as implementing the Endoscopic Third Ventriculostomy Success Score (ETVSS) (García et al., 2012; Labidi et al., 2015).

Several systematic reviews have examined ETV outcomes previously in specific pediatric populations (Kulkarni et al., 2009; Weil et al., 2016; Minta et al., 2024), in normal pressure hydrocephalus (Sohail et al., 2024), and one narrative review about clinical aspects (Yadav et al., 2021). To our knowledge, the most recent comprehensive systematic review of complications in mixed-age ETV populations was conducted by Bouras and Sgouros in 2011 (Bouras and Sgouros, 2011). Our systematic review provides updated contemporary benchmarks by incorporating 13 years of additional literature. Additionally, our analysis examines both success and complication rates, providing comprehensive outcome data for institutional benchmarking and quality assurance.

### 4.2 Risk factors

Two significant risk factors for failure of ETV were found in our series. Patients with previous shunting had a higher odds of failure compared to patients without previous shunting (OR: 17.0, 95 % CI: 4.82–70.71; p-value: <0.001). The wide confidence interval suggests imprecision of the point estimate, but the role of previous shunting as a risk factor for failure of ETV has nevertheless been shown in previous studies. Previous shunting likely reflects underlying pathophysiological complexity.

Previous neurosurgery besides shunting was also identified as a risk factor (OR: 7.33; 95 % CI: 1.94–32.0; p-value: 0.003), but point estimate imprecision was evident here as well and similarly warrants some caution in interpretation.

The fact that over half of the patients with previous neurosurgical interventions, excluding shunting, had undergone external ventricular drainage (EVD) suggests more complex and acutely presenting pathology. Given that EVD placement is typically reserved for urgent cases, this finding may contribute to improved risk stratification during the preoperative assessment. Variables such as shunt infections and revisions, obstruction through cerebral metastases, and younger age have been proposed as potential risk factors for failure of ETV (Lane and Akbari, 2022; Santamarta et al., 2004; Sacko et al., 2010b). Additionally, tools such as the aforementioned ETVSS predict outcomes and guides clinicians in patient selection (García et al., 2012; Labidi et al., 2015).

### 4.3 Limitations

There are several limitations to our study, one of which is selection bias. Patients treated with ETV are chosen based on clinical judgement, which may vary between individual neurosurgeons and centers. Our patient cohort represents those deemed most likely to benefit from ETV rather than an unselected population with obstructive hydrocephalus. Patients with significant comorbidity or poor prognosis may have been directed toward alternative treatments. This could have affected our results by inflating our success rates and underestimating complications in a broader hydrocephalus population. This limitation is also relevant for our systematic review, which is largely based on case series.

Sparse data bias (Greenland et al., 2016) constitutes another limitation to our study. Compared to high-volume centers in Africa and Asia, our series should be considered relatively small. The low sample size of our study limits our statistical power and likely prevents the detection of clinically relevant, but smaller, effects. Other possible consequences include convergence issues and inflated confidence intervals. We have attempted to mitigate this by using Firth's penalized regression, which reduces bias in parameter estimates in small sample sizes. Furthermore, there were some missing data in our material – particularly long-term follow-up. As a response, the percentage of missing data for each variable was reported to facilitate informed interpretation of our findings (Appendix 3).

### 4.4 Conclusions

In our systematic review, we found that endoscopic third ventriculostomy for obstructive hydrocephalus was successful in 73.8 % of all patients. Complications occurred in 11.6 %. Compared to our retrospective case series, we found that the results of our medium-sized neurosurgical center were comparable to those reported in the literature. The results obtained and provided in our systematic review enable other neurosurgical centers with information for internal audits.

### 4.5 Implications

We have conducted a quality assurance study that identified two risk factors which will be incorporated into the patient selection process at our center. A prospective multicenter study would be valuable to establish evidence-based patient selection criteria for adults.

## Patient consent

Patient consent was not required for this study, as described under *Ethics Approval*.

## Contributors

Christian André Helland (CAH) and Rupavathana Mahesparan (RM) jointly conceived the idea and design for the study. The data collection, statistical analysis and writing of the first drafts was done by Einar Naveen Møen (ENM). All authors contributed to the revision of subsequent versions of the manuscript.

## Ethics Approval

The study protocol was exempt from review by the Regional Ethical Committee of Western Norway as it classifies as a quality improvement study, which only requires permission from the local hospital. This arrangement is regulated by Norwegian law under The Personal Data Act (personopplysningsloven) article 6.1.e, and article 9.2.i, and the Specialist Healthcare Act (spesialisthelsetjenesteloven) §§ 3-4a.

## Data availability statement

The data collected and analyzed for our systematic review is available in Supplementary File 1. The dataset generated and analyzed for our retrospective case series is not publicly available due to the inclusion of confidential information from the included patients.

## Funding

No funding was requested or received for this study.

## Conflict of interest statement

The authors whose names are listed below certify that they have no affiliations with or involvement in any organization or entity with any financial interest (such as honoraria; educational grants; participation in speakers’ bureaus; membership, employment, consultancies, stock ownership, or other equity interest; and expert testimony or patent-licensing arrangements), or non-financial interest (such as personal or professional relationships, affiliations, knowledge or beliefs) in the subject matter or materials discussed in this manuscript.
