# Supplementary material for: Complications and risk factors of endoscopic third ventriculostomy: A 10-year single-centre study and systematic literature review
Source: Brain Spine. 2025 Jun 1;5:104291. doi: 10.1016/j.bas.2025.104291 (PMC12171757; doi:10.1016/j.bas.2025.104291)
Supplement: Multimedia component 1 [file mmc1.docx]

| Appendix 1: Data Extraction Sheet for Retrospective Analyses | | |
| --- | --- | --- |
| Area | Items | Total |
| Descriptive Characteristics | admission_date, discharge_date, age_at_surgery, diagnosis_codes, surgery_procedure_codes, surgery_duration, bmi, reason_for_hydrocephalus | 8 |
| Medical History | known_diabetes_mellitus, known_pituitary_disorder, known_vision_disorder, known_epilepsy, known_immunodeficiency, known_cardiovascular_disease, neurological_disease_besides_hydrocephalus, number_of_previous_shunts, number_of_previous_shunt_infections, other_previous_neurosurgical_interventions, anticoagulants, antihypertensives, cholesterol_lowering, smoking, alcohol | 15 |
| Quality Indicators | 30_day_reoperation, 30_day_readmission, 20_day_wound_infection, 30_day_nosocomial_infection, 30_day_other_infection, 30_day_mortality, any_complication, length_of_stay | 7 |
| 30 Day Complications | postoperative_bleeding, postoperative_usage_of_external_ventricular_drain, postoperative_pituitary_disorder, postoperative_electrolyte_disorder, postoperative_vision_disorder, postoperative_dizziness, postoperative_headache, postoperative_deep_vein_thrombosis, postoperative_strength_deficit, postoperative_epilepsy, postoperative_neurological_deficits, postoperative_pituitary_disorders, postoperative_meningitis, postoperative_CFS_leakage | 10 |
| First Follow Up Outcomes | date_first_follow_up, open_stoma_on_MRI_1, symptomatic_relief_1 | 2 |
| Last Follow Up Outcomes | date_last_follow_up, open_stoma_on_MRI_2, symptomatic_relief_2, VP_shunting, date_of_VP_shunting | 3 |

*Appendix 1. This table shows the data extraction sheet used for data collection in our retrospective analysis.*

| Appendix 2: Search Syntax in Ovid | | |
| --- | --- | --- |
| # | Search | Results |
| 1 | “endoscopic third ventriculostomy”.m_titl | 1,856 |
| 2 | “endoscopic third ventriculostomy”.mp | 4,300 |
| 3 | “hydrocephalus”.mp | 101,115 |
| 4 | “complications”.mp | 4,979,183 |
| 5 | “risk factor”.mp | 3,391,087 |
| 6 | 4 or 5 | 7,806,085 |
| 7 | 1 and 2 and 3 and 6 | 751 |
| 8 | remove duplicates from 7 | 514 |

*Appendix 2. This table shows the search strategy in detail for our systematic review.*

| Appendix 3: Missing Data | | |
| --- | --- | --- |
|  | Count (n) | Percent (%) |
| Retrospective Analysis |  |  |
| hydrocephalus etiology | 28 | 22.00 % |
| BMI | 27 | 21.30 % |
| smoking | 8 | 6.30 % |
| alcohol | 8 | 6.30 % |
| days from surgery to first follow-up | 44 | 34.60 % |
| objective relief on MRI first follow up | 48 | 37.80 % |
| subjective relief first follow up | 49 | 38.60 % |
| days from surgery to last follow up | 87 | 68.50 % |
| objective relief on MRI last follow up | 90 | 70.90 % |
| subjective relief last follow up | 91 | 71.70 % |
| Systematic Review |  |  |
| gender | 1711 | 20.35 % |
| success rates | 2170 | 25.81 % |
| complication rates | 916 | 10.89 % |
| permanent morbidity | 5867 | 69.77 % |
| mortality | 1398 | 16.63 % |

*Appendix 3. This table shows the extent of missing data in variables with any missing data in our retrospective analysis and systematic review. Missing data was largest for last follow-ups.*

| Appendix 4: Follow-Up | | | | |
| --- | --- | --- | --- | --- |
| Variable | Total (n = 127) | Male (n = 72) | Female (n = 55) | P-value |
| First Follow-Up |  |  |  |  |
| average days since surgery | 159.45 | 136.09 | 187.11 | 0.19 |
| relief on imaging (%) | 86.08 % | 88.10 % | 83.78 % | 0.75 |
| relief of symptoms (%) | 83.33 % | 82.61 % | 84.38 % | 1.00 |
| Last Follow-Up |  |  |  |  |
| average days since surgery | 815.03 | 880.57 | 726.35 | 0.54 |
| relief on imaging (%) | 81.08 % | 80.95 % | 81.25 % | 1.00 |
| relief of symptoms (%) | 77.78 % | 73.91 % | 84.62 % | 0.68 |

*Appendix 4. This table shows data from follow-up consultations in patients included in our retrospective review in total, and for patients with and without prior shunting specifically. Given the very large extent of missing data, as shown in Appendix 3, this table is likely biased and potentially misleading.*

## Appendix 5: Citation of Reports

1. One report was excluded as it was not in English (1).
2. Five reports were excluded because of dual interventions (2-6).
3. Eighty-tree reports were excluded because less than three complications were reported (7-89).
4. Sixty-four reports were included in the final review (90-153), where one report (148) was included through reverse snowballing from the last review in 2011 (154).

## References

1. Linares Torres J, Ros Lopez B, Iglesias Morono S, Ros Sanjuan A, Selfa Rodriguez A, Cerro Larrazabal L, et al. Re-Do endoscopic third ventriculostomy. Retrospective analysis of 13 patients. Neurocirugia. 2022;33(3):111-9.

2. El-Ghandour NMF, Salama MM, Ghoneim MA, Attia AM. Endoscopic third ventriculostomy for management of hydrocephalus associated with Chiari malformation type II in children. Childs Nervous System. 2023;39(6):1565-71.

3. Parikh D, Foroughi M, Nannapaneni R, Hatfield RH. Is the routine placement of a CSF reservoir following endoscopic third ventriculostomy justified. British Journal of Neurosurgery. 2009;23(5):521-3.

4. Biluts H, Admasu AK. Outcome of Endoscopic Third Ventriculostomy in Pediatric Patients at Zewditu Memorial Hospital, Ethiopia. World Neurosurgery. 2016;92:360-5.

5. O'Brien DF, Hayhurst C, Pizer B, Mallucci CL. Outcomes in patients undergoing single-trajectory endoscopic third ventriculostomy and endoscopic biopsy for midline tumors presenting with obstructive hydrocephalus. Journal of Neurosurgery. 2006;105 PEDIATRICS(SUPPL. 3):219-26.

6. Xiao B, Roth J, Udayakumaran S, Beni-Adani L, Constantini S. Placement of Ommaya reservoir following endoscopic third ventriculostomy in pediatric hydrocephalic patients: A critical reappraisal. Child's Nervous System. 2011;27(5):749-55.

7. Wang Y, Sun P, Liu Y, Du J, Zeng G. Analysis of the risk factors of post-operative seizure in pediatric patients with hydrocephalus undergoing endoscopic third ventriculostomy. Child's Nervous System. 2022;38(11):2141-8.

8. Garg AK, Suri A, Sharma BS, Shamim SA, Bal CS. Changes in cerebral perfusion hormone profile and cerebrospinal fluid flow across the third ventriculostomy after endoscopic third ventriculostomy in patients with aqueductal stenosis: a prospective study. Clinical article. Journal of Neurosurgery Pediatrics. 2009;3(1):29-36.

9. Sistiaga IL, Catalan-Uribarrena G, Perez-Fernandez S, Carrasco A, Iglesias J, Ruiz de Gopegui E, et al. Combined Predictive Model for Endoscopic Third Ventriculostomy Success in Adults and Children. World Neurosurgery. 2024;185:e721-e30.

10. Gholampour S, Bahmani M, Shariati A. Comparing the efficiency of two treatment methods of hydrocephalus: Shunt implantation and endoscopic third ventriculostomy. Basic and Clinical Neuroscience. 2019;10(3):185-98.

11. Raut R, Shams S, Scalia G, Umana GE, Ranganathan S, Rasheed M, et al. Comparison of ventriculoperitoneal shunt versus endoscopic third ventriculostomy in managing hydrocephalus due to tuberculous meningitis: A randomized controlled trial with a 30-day follow-up. Annals of Medicine and Surgery. 2024;86(2):881-5.

12. Adebayo BO, Kanu OO, Bankole OB, Ojo OA, Adetunmbi B, Morgan E. Early Outcome of Endoscopic Third Ventriculostomy With Choroid Plexus Cauterization Versus Ventriculoperitoneal Shunt as Primary Treatment of Hydrocephalus in Children With Myelomeningocele: A Prospective Cohort Study. Operative Neurosurgery. 2021;21(6):461-6.

13. Kang YS, Park EK, Kim JS, Kim DS, Thomale UW, Shim KW. Efficacy of endoscopic third ventriculostomy in old aged patients with normal pressure hydrocephalus. Neurologia i Neurochirurgia Polska. 2018;52(1):29-34.

14. Ojo OA, Bankole OB, Kanu OO, Okubadejo NU. Efficacy of endoscopic third ventriculostomy in the management of hydrocephalus in children under 2 years of age: experience from a tertiary institution in Nigeria. Nigerian journal of clinical practice. 2015;18(3):318-22.

15. Chan DYC, Tsang ACO, Ho WWS, Cheng KKF, Li LF, Tsang FCP, et al. Emergency endoscopic third ventriculostomy for blocked shunts? Univariate and multivariate analysis of independent predictors for failure. Journal of Neurosurgery. 2019;131(4):1004-10.

16. Konar S, Singha S, Shukla D, Sadashiva N, Prabhuraj AR. Endoscopic third ventriculostomy (ETV) or ventriculoperitoneal shunt (VPS) for paediatric hydrocephalus due to primary aqueductal stenosis. Child's Nervous System. 2024;40(3):685-93.

17. Atkins TG, Peters DR, Jernigan SC, Henegar MM, Van Poppel MD, Wait SD. Endoscopic Third Ventriculostomy and Endoscopic Intracranial Cyst Fenestration in an Outpatient Ambulatory Surgery Center Yields Reduced Cost But Equal Efficacy and Safety Compared with Surgery in the Hospital. World Neurosurgery. 2021;156:e160-e6.

18. Li KW, Roonprapunt C, Lawson HC, Abbott IR, Wisoff J, Epstein F, et al. Endoscopic third ventriculostomy for hydrocephalus associated with tectal gliomas. Neurosurgical Focus. 2005;18(6A):E2.

19. Smyth MD, Tubbs RS, Wellons IJC, Oakes WJ, Blount JP, Grabb PA. Endoscopic third ventriculostomy for hydrocephalus secondary to central nervous system infection or intraventricular hemorrhage in children. Pediatric Neurosurgery. 2003;39(5):258-63.

20. Oertel JMK, Mondorf Y, Baldauf J, Schroeder HWS, Gaab MR. Endoscopic third ventriculostomy for obstructive hydrocephalus due to intracranial hemorrhage with intraventricular extension: Clinical article. Journal of Neurosurgery. 2009;111(6):1119-26.

21. Obaid S, Weil AG, Rahme R, Bojanowski MW. Endoscopic third ventriculostomy for obstructive hydrocephalus due to intraventricular hemorrhage. Journal of Neurological Surgery. 2015;76(2):99-111.

22. Lipina R, Reguli S, Dolezilova V, Kuncikova M, Podesvova H. Endoscopic third ventriculostomy for obstructive hydrocephalus in children younger than 6 months of age: is it a first-choice method? Childs Nervous System. 2008;24(9):1021-7.

23. Baldauf J, Oertel J, Gaab MR, Schroeder HWS. Endoscopic third ventriculostomy for occlusive hydrocephalus caused by cerebellar infarction. Neurosurgery. 2006;59(3):539-43.

24. Raouf A, Zidan I, Mohamed E. Endoscopic third ventriculostomy for post-inflammatory hydrocephalus in pediatric patients: is it worth a try? Neurosurgical review. 2015;38(1):149-55.

25. Boschert J, Hellwig D, Krauss JK. Endoscopic third ventriculostomy for shunt dysfunction in occlusive hydrocephalus: long-term follow up and review. Journal of Neurosurgery. 2003;98(5):1032-9.

26. Massimi L, Pravata E, Tamburrini G, Gaudino S, Pettorini B, Novegno F, et al. Endoscopic third ventriculostomy for the management of Chiari I and related hydrocephalus: outcome and pathogenetic implications. Neurosurgery. 2011;68(4):950-6.

27. Salah M, Elhuseny AY, Youssef EM. Endoscopic third ventriculostomy for the management of hydrocephalus secondary to posterior fossa tumors: A retrospective study. Surgical Neurology International. 2022;13(no pagination).

28. Rei J, Pereira J, Reis C, Salvador S, Vaz R. Endoscopic Third Ventriculostomy for the Treatment of Hydrocephalus in a Pediatric Population with Myelomeningocele. World Neurosurgery. 2017;105:163-9.

29. Rezaee O, Sharifi G, Samadian M, Haddadian K, Ali-Asgari A, Yazdani M. Endoscopic third ventriculostomy for treatment of obstructive hydrocephalus. Archives of Iranian Medicine. 2007;10(4):498-503.

30. Baeza-Anton L, Martinez-Leon MI, Ros-Lopez B, Arraez-Sanchez MA. Endoscopic third ventriculostomy in children with chronic communicating congenital hydrocephalus: a single-center cohort retrospective analysis. Child's Nervous System. 2022;38(2):319-31.

31. Al-Hakim S, Schaumann A, Tietze A, Schulz M, Thomale UW. Endoscopic third ventriculostomy in children with third ventricular pressure gradient and open ventricular outlets on MRI. Child's Nervous System. 2019;35(12):2319-26.

32. Rahman MM, Khan SIMKN, Khan RA, Islam R, Sarker MH. Endoscopic third ventriculostomy in children: problems and surgical outcome: analysis of 34 cases. Chinese Neurosurgical Journal. 2021;7(1) (no pagination).

33. Choudhary A, Sobti S, Zambre S, Bhaskar S. Endoscopic Third Ventriculostomy in Failed Ventriculoperitoneal Shunt in Pediatric Population. Asian Journal of Neurosurgery. 2020;15(4):937-40.

34. Gangemi M, Maiuri F, Buonamasa S, Colella G, De Divitiis E. Endoscopic third ventriculostomy in idiopathic normal pressure hydrocephalus. Neurosurgery. 2004;55(1):129-34.

35. Fountas KN, Kapsalaki EZ, Smisson HF, Robinson JS. Endoscopic third ventriculostomy in normal pressure hydrocephalus patients. Fluids and Barriers of the CNS Conference: 8th Annual Meeting of the International Society for Hydrocephalus and Cerebrospinal Fluid Disorders, ISHCSF Cartagena Colombia. 2017;14(Supplement 1).

36. Gallo P, Szathmari A, De Biasi S, Mottolese C. Endoscopic third ventriculostomy in obstructive infantile hydrocephalus: remarks about the so-called 'unsuccessful cases'. Pediatric Neurosurgery. 2010;46(6):435-41.

37. Vindigni M, Tuniz F, Ius T, Cramaro A, Skrap M. Endoscopic third ventriculostomy in patients with secondary triventricular hydrocephalus from a haemorrhage or ischaemia in the posterior cranial fossa. Minimally Invasive Neurosurgery. 2010;53(3):106-11.

38. Singh D, Sachdev V, Singh AK, Sinha S. Endoscopic third ventriculostomy in post-tubercular meningitic hydrocephalus: a preliminary report. Minimally Invasive Neurosurgery. 2005;48(1):47-52.

39. Brichtova E, Chlachula M, Hrbac T, Lipina R. Endoscopic third ventriculostomy in previously shunted children. Minimally Invasive Surgery. 2013;2013:584567.

40. Hailong F, Guangfu H, Haibin T, Hong P, Yong C, Weidong L, et al. Endoscopic third ventriculostomy in the management of communicating hydrocephalus: A preliminary study. Clinical article. Journal of Neurosurgery. 2008;109(5):923-30.

41. Feng H, Huang G, Liao X, Fu K, Tan H, Pu H, et al. Endoscopic third ventriculostomy in the management of obstructive hydrocephalus: An outcome analysis. Journal of Neurosurgery. 2004;100(4):626-33.

42. Fernandez LA, Mastrapa TL, De Jongh Cobo E, Diaz Alvarez M. Endoscopic third ventriculostomy in the treatment of 76 Hydrocephalic patients. Follow up for 8 years. Child's Nervous System. 2012;28(9):1636-7.

43. Dong V, Ly N, Teo C. Endoscopic third ventriculostomy in the treatment of childhood non-communicating hydrocephalus: Experience in VietDuc Teaching Hospital. Child's Nervous System. 2012;28(9):1591-2.

44. Yadav YR, Parihar V, Agrawal M, Bhatele PR. Endoscopic third ventriculostomy in tubercular meningitis with hydrocephalus. Neurology India. 2011;59(6):855-60.

45. Ramakrishna Murty TV, Basha SA, Rao TN. Endoscopic third ventriculostomy intraoperative complications. Journal of Pediatric Neurosciences. 2010;5(2):178-9.

46. Debs LH, Rahimi SY, Rutkowski MJ, Macomson SD. Endoscopic third ventriculostomy may decrease shunt-dependency in patients with post-hemorrhagic hydrocephalus following aneurysmal subarachnoid hemorrhage. Interdisciplinary Neurosurgery: Advanced Techniques and Case Management. 2021;26(no pagination).

47. Frisoli F, Kakareka M, Cole KA, Waanders AJ, Storm PB, Lang SS. Endoscopic third ventriculostomy prior to resection of posterior fossa tumors in children. Child's Nervous System. 2019;35(5):789-94.

48. El-Ghandour NM. Endoscopic third ventriculostomy versus ventriculoperitoneal shunt in the treatment of obstructive hydrocephalus due to posterior fossa tumors in children. Childs Nervous System. 2011;27(1):117-26.

49. Buxton N, Cartmill M, Vloeberghs M. Endoscopic third ventriculostomy: outcome analysis of 100 consecutive procedures. Neurosurgery. 1999;45(4):957-9.

50. Longatti PL, Fiorindi A, Martinuzzi A. Failure of endoscopic third ventriculostomy in the treatment of idiopathic normal pressure hydrocephalus. Minimally Invasive Neurosurgery. 2004;47(6):342-5.

51. Elbabaa SK, Gildehaus AM, Pierson MJ, Albers JA, Vlastos EJ. First 60 fetal in-utero myelomeningocele repairs at Saint Louis Fetal Care Institute in the post-MOMS trial era: hydrocephalus treatment outcomes (endoscopic third ventriculostomy versus ventriculo-peritoneal shunt). Child's Nervous System. 2017;33(7):1157-68.

52. Sharafat S, Khan Z, Azam F, Ali M. Frequency of success and complications of primary endoscopic third ventriculostomy in infants with obstructive hydrocephalous. Pakistan Journal of Medical Sciences. 2022;38(1):267-70.

53. Tisell M, Almstrom O, Stephensen H, Tullberg M, Wikkelso C. How effective is endoscopic third ventriculostomy in treating adult hydrocephalus caused by primary aqueductal stenosis? Neurosurgery. 2000;46(1):104-10; discussion 10-1.

54. Kim J, Patel VJ, El Ahmadieh TY, Olson DM, Swift DM. Hydrocephalus in achondroplasia: efficacy of endoscopic third ventriculostomy. Journal of Neurosurgery: Pediatrics. 2022;29(3):268-75.

55. Fritsch MJ, Doerner L, Kienke S, Mehdorn HM. Hydrocephalus in children with posterior fossa tumors: role of endoscopic third ventriculostomy. Journal of Neurosurgery. 2005;103(1 Suppl):40-2.

56. Wiewrodt D, Schumacher R, Wagner W. Hygromas after endoscopic third ventriculostomy in the first year of life: Incidence, management and outcome in a series of 34 patients. Child's Nervous System. 2008;24(1):57-63.

57. Paidakakos N, Borgarello S, Naddeo M. Indications for endoscopic third ventriculostomy in normal pressure hydrocephalus. Hydrocephalus: Selected Papers from the International Workshop in Crete, 2010. 2012;Acta Neurochirurgica, Supplementum.(113):123-7.

58. Idowu O, Doherty A, Tiamiyu O. Initial experience with endoscopic third ventriculostomy in Nigeria, West Africa. Childs Nervous System. 2008;24(2):253-5; discussion 7.

59. Hayhurst C, Osman-Farah J, Das K, Mallucci C. Initial management of hydrocephalus associated with Chiari malformation Type I-syringomyelia complex via endoscopic third ventriculostomy: an outcome analysis. Journal of Neurosurgery. 2008;108(6):1211-4.

60. Greenfield JP, Hoffman C, Kuo E, Christos PJ, Souweidane MM. Intraoperative assessment of endoscopic third ventriculostomy success: Clinical article. Journal of Neurosurgery: Pediatrics. 2008;2(5):298-303.

61. Baroncini M, Kuchcinski G, Le Thuc V, Bourgeois P, Leroy HA, Baille G, et al. Is endoscopic third ventriculostomy safe and efficient in the treatment of obstructive chronic hydrocephalus in adults? A prospective clinical and MRI study. Acta Neurochirurgica. 2019;161(7):1353-60.

62. Fukuhara T, Shimizu T, Namba Y. Limited efficacy of endoscopic third ventriculostomy for hydrocephalus following aneurysmal subarachnoid hemorrhage. Neurologia Medico-Chirurgica. 2009;49(10):449-55.

63. Romeo A, Naftel RP, Griessenauer CJ, Reed GT, Martin R, Shannon CN, et al. Long-term change in ventricular size following endoscopic third ventriculostomy for hydrocephalus due to tectal plate gliomas. Journal of Neurosurgery Pediatrics. 2013;11(1):20-5.

64. Wellons JC, 3rd, Tubbs RS, Banks JT, Grabb B, Blount JP, Oakes WJ, et al. Long-term control of hydrocephalus via endoscopic third ventriculostomy in children with tectal plate gliomas. Neurosurgery. 2002;51(1):63-7; discussion 7-8.

65. Stovell MG, Zakaria R, Ellenbogen JR, Gallagher MJ, Jenkinson MD, Hayhurst O, et al. Long-term follow-up of endoscopic third ventriculostomy performed in the pediatric population. Journal of Neurosurgery: Pediatrics. 2016;17(6):734-8.

66. Oertel J, Vulcu S, Eickele L, Wagner W, Cinalli G, Rediker J. Long-Term Follow-Up of Repeat Endoscopic Third Ventriculostomy in Obstructive Hydrocephalus. World Neurosurgery. 2017;99:556-65.

67. Faggin R, Calderone M, Denaro L, Meneghini L, d'Avella D. Long-term operative failure of endoscopic third ventriculostomy in pediatric patients: the role of cine phase-contrast MR imaging. Neurosurgical Focus. 2011;30(4):E1.

68. Takeshige N, Uchikado H, Yoshitake H, Negoto T, Yoshitomi M, Sakata K, et al. Long-term outcomes of endoscopic third ventriculostomy for Blake's pouch cyst in adults. Clinical Neurology and Neurosurgery. 2021;200(no pagination).

69. Teo C, Jones R. Management of hydrocephalus by endoscopic third ventriculostomy in patients with myelomeningocele. Pediatric Neurosurgery. 1996;25(2):57-63; discussion

70. Chimaliro S, Hara C, Kamalo P. Mortality and complications 1 year after treatment of hydrocephalus with endoscopic third ventriculostomy and ventriculoperitoneal shunt in children at Queen Elizabeth Central Hospital, Malawi. Acta Neurochirurgica. 2023;165(1):61-9.

71. Kramer U, Kanner AA, Siomin V, Harel S, Constantini S. No evidence of epilepsy following endoscopic third ventriculostomy: A short-term follow-up. Pediatric Neurosurgery. 2001;34(3):121-3.

72. Lam S, Harris DA, Lin Y, Rocque BG, Ham S, Pan IW. Outcomes of endoscopic third ventriculostomy in adults. Journal of Clinical Neuroscience. 2016;31:166-71.

73. Rennert RC, Brandel MG, Srinivas S, Prajapati D, Al Jammal OM, Brown NJ, et al. Palliative endoscopic third ventriculostomy for pediatric primary brain tumors: A single-institution case series. Journal of Neurosurgery: Pediatrics. 2021;28(4):387-94.

74. Chen CC, Kasper E, Warnke P. Palliative stereotactic-endoscopic third ventriculostomy for the treatment of obstructive hydrocephalus from cerebral metastasis. Surgical neurology international. 2011;2:76.

75. Morelli D, Pirotte B, Lubansu A, Detemmerman D, Aeby A, Fricx C, et al. Persistent hydrocephalus after early surgical management of posterior fossa tumors in children: is routine preoperative endoscopic third ventriculostomy justified? Journal of neurosurgery. 2005;103(3 Suppl):247-52.

76. Fabiano AJ, Leonardo J, Grand W. Posterior cerebral artery P1 segment at the stoma during endoscopic third ventriculostomy in adults. Journal of Neurology, Neurosurgery & Psychiatry. 2010;81(4):374-8.

77. Guida L, Grenier F, Beccaria K, Benichi S, James S, Paternoster G, et al. Predicting endoscopic third ventriculostomy success in pediatric shunt dysfunction: a monocentric retrospective case-series of 70 consecutive children. Child's Nervous System. 2023;39(5):1389.

78. Kulkarni AV, Drake JM, Kestle JR, Mallucci CL, Sgouros S, Constantini S. Predicting who will benefit from endoscopic third ventriculostomy compared with shunt insertion in childhood hydrocephalus using the ETV Success Score. Journal of Neurosurgery Pediatrics. 2010;6(4):310-5.

79. Woodworth GF, See A, Bettegowda C, Batra S, Jallo GI, Rigamonti D. Predictors of surgery-free outcome in adult endoscopic third ventriculostomy. World Neurosurgery. 2012;78(3-4):312-7.

80. Azab W, Al-Sheikh T, Yahia A. Preoperative endoscopic third ventriculostomy in children with posterior fossa tumors: an institution experience. Turkish Neurosurgery. 2013;23(3):359-65.

81. Woodworth G, McGirt MJ, Thomas G, Williams MA, Rigamonti D. Prior CSF shunting increases the risk of endoscopic third ventriculostomy failure in the treatment of obstructive hydrocephalus in adults. Neurological Research. 2007;29(1):27-31.

82. Aranha A, Choudhary A, Bhaskar S, Gupta LN. A Randomized Study Comparing Endoscopic Third Ventriculostomy versus Ventriculoperitoneal Shunt in the Management of Hydrocephalus Due to Tuberculous Meningitis. Asian Journal of Neurosurgery. 2018;13(4):1140-7.

83. Liu S, Chen P, Yang H, Xie T, Liu T, Li C, et al. Role of endoscopic third ventriculostomy in patients undergoing resection of pulvinar area lesions: Preliminary clinical results. Journal of Clinical Neuroscience. 2023;117:61-7.

84. Beuriat PA, Szathmari A, Grassiot B, Plaisant F, Rouselle C, Mottolese C. Role of endoscopic third ventriculostomy in the management of myelomeningocele related hydrocephalus. Child's Nervous System. 2015;31(10):1935.

85. Di Rocco F, Juca CE, Arnaud E, Renier D, Sainte-Rose C. The role of endoscopic third ventriculostomy in the treatment of hydrocephalus associated with faciocraniosynostosis. Journal of Neurosurgery Pediatrics. 2010;6(1):17-22.

86. Schulz M, Spors B, Thomale UW. Stented endoscopic third ventriculostomy-indications and results. Childs Nervous System. 2015;31(9):1499-507.

87. Chugh A, Husain M, Gupta RK, Ojha BK, Chandra A, Rastogi M. Surgical outcome of tuberculous meningitis hydrocephalus treated by endoscopic third ventriculostomy: prognostic factors and postoperative neuroimaging for functional assessment of ventriculostomy. Journal of Neurosurgery Pediatrics. 2009;3(5):371-7.

88. Baldauf J, Fritsch MJ, Oertel J, Gaab MR, Schroder H. Value of endoscopic third ventriculostomy instead of shunt revision. Minimally Invasive Neurosurgery. 2010;53(4):159-63.

89. Gonda DD, Kim TE, Warnke PC, Kasper EM, Carter BS, Chen CC. Ventriculoperitoneal shunting versus endoscopic third ventriculostomy in the treatment of patients with hydrocephalus related to metastasis. Surgical neurology international. 2012;3:97.

90. Meling TR, Tiller C, Due-Tonnessen BJ, Egge A, Eide PK, Froslie KF, et al. Audits can improve neurosurgical practice--illustrated by endoscopic third ventriculostomy. Pediatric Neurosurgery. 2007;43(6):482-7.

91. Kawsar KA, Haque MR, Chowdhury FH. Avoidance and management of perioperative complications of endoscopic third ventriculostomy: The Dhaka experience. Journal of Neurosurgery. 2015;123(6):1414-9.

92. Zwimpfer TJ, Salterio N, Williams MA, Holubkov R, Katzen H, Luciano MG, et al. Cognitive and gait outcomes after primary endoscopic third ventriculostomy in adults with chronic obstructive hydrocephalus. Journal of Neurosurgery. 2022;136(3):887-94.

93. Prajapati HP, Ansari MA, Jaiswal M. Comparative Outcome Analysis of Endoscopic Third Ventriculostomy and Ventriculoperitoneal Shunt Surgery in Pediatric Hydrocephalus: An Experience of a Tertiary Care Center. Asian Journal of Neurosurgery. 2022;17(2):227-34.

94. Ersahin Y, Arslan D. Complications of endoscopic third ventriculostomy. Child's Nervous System. 2008;24(8):943-8.

95. Schroeder HWS, Niendorf WR, Gaab MR. Complications of endoscopic third ventriculostomy. Journal of Neurosurgery. 2002;96(6):1032-40.

96. Hader WJ, Walker RL, Myles ST, Hamilton M. Complications of endoscopic third ventriculostomy in previously shunted patients. Neurosurgery. 2008;63(1 SUPPL.):ONS168-ONS74.

97. Uche EO, Okorie C, Iloabachie I, Amuta DS, Uche NJ. Endoscopic third ventriculostomy (ETV) and ventriculoperitoneal shunt (VPS) in non-communicating hydrocephalus (NCH): comparison of outcome profiles in Nigerian children. Childs Nervous System. 2018;34(9):1683-9.

98. Tabakow P, Weiser A, Burzynska M, Blauciak P. Endoscopic third ventriculostomy before surgery of third ventricle and posterior fossa tumours decreases the risk of secondary hydrocephalus and early postoperative complications. Neurosurgical Review. 2022;45(1):771-81.

99. Gangemi M, Donati P, Maiuri F, Longatti P, Godano U, Mascari C. Endoscopic third ventriculostomy for hydrocephalus. Minimally Invasive Neurosurgery. 1999;42(3):128-32.

100. Ali M, Usman M, Khan Z, Khan KM, Hussain R, Khanzada K. Endoscopic third ventriculostomy for obstructive hydrocephalus. Journal of the College of Physicians and Surgeons Pakistan. 2013;23(5):338-41.

101. Khan MB, Riaz M, Enam SA. Endoscopic third ventriculostomy for obstructive hydrocephalus: Outcome analysis of 120 consecutively treated patients from a developing country. International Journal of Surgery. 2016;26:69-72.

102. Ray P, Jallo GI, Kim RYH, Kim BS, Wilson S, Kothbauer K, et al. Endoscopic third ventriculostomy for the treatment of hydrocephalus: An alternative to shunting. Journal of Pediatric Neurology. 2006;4(4):221-32.

103. Isaacs AM, Bezchlibnyk YB, Yong H, Koshy D, Urbaneja G, Hader WJ, et al. Endoscopic third ventriculostomy for treatment of adult hydrocephalus: long-term follow-up of 163 patients. Neurosurgical focus. 2016;41(3):E3.

104. Ray P, Jallo GI, Kim RY, Kim BS, Wilson S, Kothbauer K, et al. Endoscopic third ventriculostomy for tumor-related hydrocephalus in a pediatric population. Neurosurgical focus. 2005;19(6):E8.

105. Grand W, Leonardo J, Chamczuk AJ, Korus AJ. Endoscopic third ventriculostomy in 250 adults with hydrocephalus: Patient selection, outcomes, and complications. Neurosurgery. 2015;78(1):109-19.

106. Gorayeb RP, Cavalheiro S, Zymberg ST. Endoscopic third ventriculostomy in children younger than 1 year of age. Journal of Neurosurgery. 2004;100(5 SUPPL.):427-9.

107. Navarro R, Gil-Parra R, Reitman AJ, Olavarria G, Grant JA, Tomita T. Endoscopic third ventriculostomy in children: Early and late complications and their avoidance. Child's Nervous System. 2006;22(5):506-13.

108. Kulkarni AV, Riva-Cambrin J, Holubkov R, Browd SR, Cochrane DD, Drake JM, et al. Endoscopic third ventriculostomy in children: Prospective, multicenter results from the Hydrocephalus Clinical Research Network. Journal of Neurosurgery: Pediatrics. 2016;18(4):423-9.

109. Chowdhary S, Panigrahi P, Yadav MK, Sharma SP. Endoscopic Third Ventriculostomy in Infants Less than One Year of Age: A Short Series of 14 Cases. Pediatric Neurosurgery. 2021;56(2):105-9.

110. Sarmast A, Khursheed N, Ramzan A, Shaheen F, Wani A, Singh S, et al. Endoscopic Third Ventriculostomy in Noncommunicating Hydrocephalus: Report on a Short Series of 53 Children. Asian Journal of Neurosurgery. 2019;14(1):35-40.

111. Siomin V, Cinalli G, Grotenhuis A, Golash A, Oi S, Kothbauer K, et al. Endoscopic third ventriculostomy in patients with cerebrospinal fluid infection and/or hemorrhage. Journal of Neurosurgery. 2002;97(3):519-24.

112. Melikian A, Korshunov A. Endoscopic third ventriculostomy in patients with malfunctioning CSF-Shunt. World Neurosurgery. 2010;74(4-5):532-7.

113. Furtado LMF, da Costa Val Filho JA, Holliday JB, da Silva Costa J, de Matos MA, Nascimento VAM, et al. Endoscopic third ventriculostomy in patients with myelomeningocele after shunt failure. Child's Nervous System. 2020;36(12):3047-52.

114. Rocque BG, Jensen H, Reeder RW, Kulkarni AV, Pollack IF, Wellons JC, et al. Endoscopic third ventriculostomy in previously shunt-treated patients. Journal of Neurosurgery: Pediatrics. 2022;30(4):428-36.

115. Ruggiero C, Cinalli G, Spennato P, Aliberti F, Cianciulli E, Trischitta V, et al. Endoscopic third ventriculostomy in the treatment of hydrocephalus in posterior fossa tumors in children. Childs Nervous System. 2004;20(11-12):828-33.

116. Faquini IV, Fonseca RB, Correia AO, Cezar Junior AB, de Carvalho Junior EV, de Almeida NS, et al. Endoscopic third ventriculostomy in the treatment of hydrocephalus: A 20-year retrospective analysis of 209 consecutive cases. Surgical Neurology International. 2021;12(no pagination).

117. Kontojannis V, Papadopoulos E, Ydreos J, Isaakidis D, Gavra MM, Boviatsis EJ. Endoscopic Third Ventriculostomy: A Single-Center Experience in Greece. The Surgery Journal. 2020;6(2):e71-e6.

118. Brockmeyer D, Abtin K, Carey L, Walker ML. Endoscopic third ventriculostomy: An outcome analysis. Pediatric Neurosurgery. 1998;28(5):236-40.

119. O'Brien DF, Javadpour M, Collins DR, Spennato P, Mallucci CL. Endoscopic third ventriculostomy: an outcome analysis of primary cases and procedures performed after ventriculoperitoneal shunt malfunction. Journal of neurosurgery. 2005;103(5 Suppl):393-400.

120. Vemula RCV, Prasad BCM, Kumar K. Endoscopic Third Ventriculostomy: Our Experience of Consecutive 50 Cases at a Tertiary Care Center. Indian Journal of Neurosurgery. 2022;11(1):39-48.

121. Sacko O, Boetto S, Lauwers-Cances V, Dupuy M, Roux FE. Endoscopic third ventriculostomy: Outcome analysis in 368 procedures: Clinical article. Journal of Neurosurgery: Pediatrics. 2010;5(1):68-74.

122. Hopf NJ, Grunert P, Fries G, Resch KDM, Perneczky A. Endoscopic third ventriculostomy: Outcome analysis of 100 consecutive procedures. Neurosurgery. 1999;44(4):795-806.

123. Aref M, Martyniuk A, Nath S, Koziarz A, Badhiwala J, Algird A, et al. Endoscopic Third Ventriculostomy: Outcome Analysis of an Anterior Entry Point. World Neurosurgery. 2017;104:554-9.

124. Rahme R, Rahme RJ, Hourani R, Moussa R, Nohra G, Okais N, et al. Endoscopic third ventriculostomy: The lebanese experience. Pediatric Neurosurgery. 2009;45(5):361-7.

125. Naftel RP, Reed GT, Kulkarni AV, Wellons IJC. Evaluating the Children's Hospital of Alabama endoscopic third ventriculostomy experience using the endoscopic third ventriculostomy success score: An external validation study - Clinical article. Journal of Neurosurgery: Pediatrics. 2011;8(5):494-501.

126. Goel A, Galloway L, Abualsaud S, Chowdhury Y, Gan P, Flint G, et al. Factors affecting endoscopic third ventriculostomy success in adults. Acta Neurochirurgica. 2023;165(12):4021-9.

127. Bisht A, Suri A, Bansal S, Chandra PS, Kumar R, Singh M, et al. Factors affecting surgical outcome of endoscopic third ventriculostomy in congenital hydrocephalus. Journal of Clinical Neuroscience. 2014;21(9):1483-9.

128. Warf BC. Hydrocephalus in Uganda: the predominance of infectious origin and primary management with endoscopic third ventriculostomy. Journal of neurosurgery. 2005;102(1 Suppl):1-15.

129. Chan AK, McGovern RA, Zacharia BE, Mikell CB, Bruce SS, Sheehy JP, et al. Inferior short-term safety profile of endoscopic third ventriculostomy compared with ventriculoperitoneal shunt placement for idiopathic normal-pressure hydrocephalus: A population-based study. Neurosurgery. 2013;73(6):951-61.

130. Van Beijnum J, Hanlo PW, Fischer K, Majidpour MM, Kortekaas MF, Verdaasdonk RM, et al. Laser-assisted endoscopic third ventriculostomy: Long-term results in a series of 202 patients. Neurosurgery. 2008;62(2):437-43.

131. Gangemi M, Mascari C, Maiuri F, Godano U, Donati P, Longatti PL. Long-term outcome of endoscopic third ventriculostomy in obstructive hydrocephalus. Minimally Invasive Neurosurgery. 2007;50(5):265-9.

132. Waqar M, Ellenbogen JR, Stovell MG, Al-Mahfoudh R, Mallucci C, Jenkinson MD. Long-Term Outcomes of Endoscopic Third Ventriculostomy in Adults. World Neurosurgery. 2016;94:386-93.

133. Kadrian D, Van Gelder J, Florida D, Jones R, Vonau M, Teo C, et al. Long-term reliability of endoscopic third ventriculostomy. Neurosurgery. 2005;56(6):1271-8.

134. Kadrian D, van Gelder J, Florida D, Jones R, Vonau M, Teo C, et al. Long-term reliability of endoscopic third ventriculostomy. Neurosurgery. 2008;62 Suppl 2:614-21.

135. Sankey EW, Goodwin CR, Jusue-Torres I, Elder BD, Hoffberger J, Lu J, et al. Lower rates of symptom recurrence and surgical revision after primary compared with secondary endoscopic third ventriculostomy for obstructive hydrocephalus secondary to aqueductal stenosis in adults. Journal of Neurosurgery. 2016;124(5):1413-20.

136. Bhatia R, Tahir M, Chandler CL. The management of hydrocephalus in children with posterior fossa tumours: The role of pre-resectional endoscopic third ventriculostomy. Pediatric Neurosurgery. 2009;45(3):186-91.

137. Sainte-Rose C, Cinalli G, Roux FE, Maixner R, Chumas PD, Mansour M, et al. Management of hydrocephalus in pediatric patients with posterior fossa tumors: the role of endoscopic third ventriculostomy. Journal of Neurosurgery. 2001;95(5):791-7.

138. Ul Haq N, Ishaq M, Jalal A. Outcome Comparison of Endoscopic Third Ventriculostomy Versus Ventriculoperitoneal Shunt in Obstructive Hydrocephalus. Pakistan Journal of Medical and Health Sciences. 2022;16(2):956-8.

139. Idowu OE, Falope LO, Idowu AT. Outcome of endoscopic third ventriculostomy and Chhabra shunt system in noncommunicating non-tumor childhood hydrocephalus. Journal of Pediatric Neurosciences. 2009;4(2):66-9.

140. Santamarta D, Diaz Alvarez A, Goncalves JM, Hernandez J. Outcome of endoscopic third ventriculostomy. Results from an unselected series with noncommunicating hydrocephalus. Acta Neurochirurgica. 2005;147(4):377-82.

141. Arslan A, Olguner SK, Acik V, Istemen I, Arslan B, Okten AI, et al. The Outcomes of Endoscopic Third Ventriculostomy in the Treatment of Hydrocephalus: 317 Pediatric and Adult Cases. Turkish neurosurgery. 2021;31(5):686-93.

142. Ujjan BU, Nawaz S, Anwer MS, Rehman S, Kamran M, Hassan N. Prevalence and Pattern of Initial Complications Following Endoscopic Third Ventriculostomy for Hydrocephalus Obstructor. Pakistan Journal of Medical and Health Sciences. 2022;16(12):570-2.

143. Goyal P, Srivastava C, Ojha BK, Singh SK, Chandra A, Garg RK, et al. A randomized study of ventriculoperitoneal shunt versus endoscopic third ventriculostomy for the management of tubercular meningitis with hydrocephalus. Child's Nervous System. 2014;30(5):851-7.

144. Fukuhara T, Vorster SJ, Luciano MG. Risk factors for failure of endoscopic third ventriculostomy for obstructive hydrocephalus. Neurosurgery. 2000;46(5):1100-11.

145. Jenkinson MD, Hayhurst C, Al-Jumaily M, Kandasamy J, Clark S, Mallucci CL. The role of endoscopic third ventriculostomy in adult patients with hydrocephalus: Clinical article. Journal of Neurosurgery. 2009;110(5):861-6.

146. Cinalli G, Salazar C, Mallucci C, Yada JZ, Zerah M, Sainte-Rose C. The role of endoscopic third ventriculostomy in the management of shunt malfunction. Neurosurgery. 1998;43(6):1323-7; discussion 7-9.

147. Vogel TW, Bahuleyan B, Robinson S, Cohen A. The role of endoscopic third ventriculostomy in the treatment of hydrocephalus. Journal of Neurosurgery. 2013;119(2):A546.

148. Grunert P, Charalampaki P, Hopf N, Filippi R. The role of third ventriculostomy in the management of obstructive hydrocephalus. Minim Invasive Neurosurg. 2003;46(1):16-21.

149. Shinde S, Kotecha N, Ansari I, Patel S. Study of endoscopic third ventriculostomy (ETV) versus ventriculoperitoneal shunt (VP shunt) in patients with hydrocephalus at a tertiary hospital. Journal of Cardiovascular Disease Research. 2023;14(4):2628-32.

150. Dusick JR, McArthur DL, Bergsneider M. Success and complication rates of endoscopic third ventriculostomy for adult hydrocephalus: a series of 108 patients. Surgical Neurology. 2008;69(1):5-15.

151. de Boorder T, Brouwers HB, Noordmans HJ, Woerdeman PA, Han KS, Verdaasdonk RM. Thulium laser-assisted endoscopic third ventriculostomy: Determining safe laser settings using in vitro model and 2 year follow-up results in 106 patients. Lasers in Surgery and Medicine. 2018;50(6):629-35.

152. Lu W, Du A, Zheng X. Treatment of post-thalamic hemorrhage hydrocephalus: ventriculoperitoneal shunt or endoscopic third ventriculostomy? A retrospective observational study. BMC Neurology. 2024;24(1):365.

153. Jung JH, Chai YH, Jung S, Kim IY, Jang WY, Moon KS, et al. Visual outcome after endoscopic third ventriculostomy for hydrocephalus. Child's Nervous System. 2018;34(2):247-55.

154. Bouras T, Sgouros S. Complications of endoscopic third ventriculostomy. J Neurosurg Pediatr. 2011;7(6):643-9.
